# Determination of genetic variation within the *DYRK2* gene and its associations with milk traits in cattle

**DOI:** 10.5194/aab-63-315-2020

**Published:** 2020-09-09

**Authors:** Cui Mao, Xing Ju, Haijian Cheng, Xixia Huang, Fugui Jiang, Yuni Yao, Xianyong Lan, Enliang Song

**Affiliations:** 1 Shandong Key Laboratory of Animal Disease Control and Breeding, Institute of Animal Science and Veterinary Medicine, Shandong Academy of Agricultural Sciences, Jinan, 250000, China; 2 College of Animal Science and Technology, Xinjiang Agricultural University, Urumqi, Xinjiang, 830000, China; 3 Key Laboratory of Animal Genetics, Breeding and Reproduction of Shaanxi Province, College of Animal Science and Technology, Northwest A&F University, Yangling, Shaanxi, 712100, China; 4 College of Life Sciences, Shandong Normal University, Jinan, Shandong, 250000, China

## Abstract

To speed up the progress of marker-assisted selection (MAS) in cattle breeding, the dual-specificity tyrosine phosphorylation-regulated kinase 2 (*DYRK2*), cadherin 2 (*CDH2*), and kinesin family member 1A (*KIF1A*) genes were chosen based on our pervious genome-wide association study (GWAS) analysis results. *DYRK2* is a kinase that may participate in cell growth and/or development; it shows phosphorylation activity toward serine, threonine, and tyrosine fragments of proteins, and it is different from other protein kinases. The *CDH2* gene encodes a classic cadherin, which is a member of the cadherin superfamily. The protein encoded by *KIF1A* is a member of the kinesin family and plays a role in the transportation of membrane organelles along axon microtubules. We detected insertion/deletion (InDel) variation in these three candidate genes in 438 individual cattle (Xinjiang Brown cattle and Wagyu × Luxi crossbreed cattle). Only *DYRK2*-P3-11 bp was polymorphic and genotyped. The polymorphism information content of *DYRK2*-P3-11 bp was 0.336. Correlation analyses showed that InDel polymorphism was significantly associated with six different milk traits. These findings may aid future analyses of InDel genotypes in cattle breeds, and speed up the progress of MAS in cattle breeding.

## 1 Introduction

Cattle are economically significant livestock. In China, Xinjiang Brown cattle (XJBC) and Wagyu × Luxi cross cattle (WLC) are high-quality domestic breeds. XJBC are an improved breed of local Chinese yellow cattle, which is obtained by crossing Chinese yellow with Brown Swiss cattle, Alatuowu cattle, and Kostroma cattle (Lin et al., 2010; Li et al., 2018). WLC are a new high-grade beef breed obtained by using modern biotechnology combined with conventional breeding techniques after 10 years of relentless effort (Wang et al., 2015). However, data on the association between their genetic background and milk traits are limited.

Milk traits are an important phenotype of cattle (Andersson et al., 2001). To have better milk production, genetic variation between cattle varieties and between major genes could be appropriately used to establish breeding programs (K. Wang et al., 2018). Genome-wide sequencing and association studies (GWASs) have been used to research genetic variation correlated with milk traits. However, many of the potential genes affecting milk traits have not been fully confirmed (Lai et al., 2016; Mota et al., 2017). To solve this problem, GWAS analyses have been used to screen large livestock populations.

Based on previous GWASs on XJBC (Zhou et al., 2019), we analyzed dual-specificity tyrosine phosphorylation-regulated kinase 2 (*DYRK2*), cadherin 2 (*CDH2*), and kinesin family member 1A (*KIF1A*) genes. *DYRK2* is a kinase that may participate in cell growth and/or development. It shows phosphorylation activity toward serine, threonine, and tyrosine fragments of proteins, and it is different from other protein kinases. As a novel phosphorylation-regulated kinase, *DYRK2* induces the phosphorylation of c-Myc in cancer cells and controls p53 by phosphorylation at Ser46 in response to DNA damage (Maddika et al., 2009; Taira et al., 2012). It plays a role in breast development (Tolleson et al., 2017). The *CDH2* gene encodes a classic cadherin, a member of the cadherin superfamily (Kumari et al., 2018). The protein encoded by *KIF1A* is a member of the kinesin family, and it plays a role in the transportation of membrane organelles along axon microtubules (Lee et al., 2003).

XJBC and WLC represent important scientific achievements in China; therefore, it is important to further develop and promote these two breeds. However, traditional breeding methods are costly. Genetic molecular markers such as insertion/deletion (InDel) variation, copy number variation (CNV), and single-nucleotide polymorphisms (SNP) are widely used to assess gene function (Yang et al., 2017; Chen et al., 2019). InDel markers, in particular, are inexpensive, a time-saver, and convenient (Jander et al., 2002; Yang et al., 2017; Ju et al., 2020). Many studies have reported InDels in several genes in cattle (Meo et al., 2005), however, InDel variation in *DYRK2*, *CHD2*, and *KIF1A* in XJBC and WLC have not been detected. Therefore, in this study, 438 individuals representing these two cattle breeds were collected to detect and evaluate InDel variation and any correlations with milk traits. Our findings may aid the future assessment and use of genetic variants associated with phenotypic traits to improve cattle farming.

## 2 Materials and methods

### 2.1 Ethics statement

All experimental animals were raised and used in accordance with local policies and animal welfare laws, and all the experimental procedures were approved by the Faculty Animal Policy and Welfare Committee of Shandong Academy of Agricultural Sciences (FAPWC-SDAAS) and Xinjiang Agricultural University (FAPWC-XJAU).

### 2.2 Animal samples and genomic DNA collection

In all, 438 individuals of the XJBC (n=388, Xinjiang Province) and WLC (n=50, Shandong Province) cattle breeds were obtained. The following phenotypic characteristics were recorded for each breed: 305 d milk yield (305M), milk fat percentage (FP), milk protein percentage (PP), milk fat yield (FY), milk protein yield (PY), and somatic cell score (SCS) (Peng et al., 2019). DNA samples were extracted from white blood cells using phenolic chloroform extraction (frozen at -80 ${}^{\circ }$C) (Zhang et al., 2015). After measurement of DNA using a Nanodrop 1000 spectrophotometer (Waltham, MA, USA), the concentrations of all DNA samples were diluted to 50 ng µL${}^{-1}$ and temporarily stored at 4 ${}^{\circ }$C (Lan et al., 2013).

### 2.3 InDel loci detection and DNA sequencing

According to the whole sequence of bovine *DYRK2* (GenBank no. NC_037332.1), *CDH2* (GenBank no. NC_037351.1), *KIF1A* (GenBank no. NC_037330.1) genes, a total of 11 potential InDel variants located in 5${}^{\prime }$ untranslated regions, 3${}^{\prime }$ untranslated regions, and introns were found in the Ensembl database (https://asia.ensembl.org/index.html, last access: 15 April 2019). Relevant primers were designed using Primer Premier software 5.0 (Premier Biosoft International, Palo Alto, CA, USA) to reference the bovine *CDH2* gene sequence, bovine *DYRK2* gene sequence, and bovine *KIF1A* gene sequence (Table 1).

**Table 1:** PCR primer sequences of three genes for amplification.

Genes	Loci	Primer sequences (5${}^{\prime }$–3${}^{\prime }$)	T (${}^{\circ }$C)	Product sizes (bp)	Region	Notes
*DYRK2*	P1-D1	F: GTTGTGGTCTCATCTGGCTC	55.9	121/114	3${}^{\prime }$ UTR	InDel detecting
		R: AAGGGCACAATTTCTCCTCTACTG	56.7			
	P2-D2	F: AGGTCATTTAGGCTCAGCATTTT	54.1	114/106	Intron 2	
		R: CTCACACTGCCCTTGACTTTG	56.5			
	P3-D3	F: ACATACGTTCTTCCATTCAGCAG	54.8	195/184	Intron 2	g46127045_
		R: AGGGTGGGGTCACCTCTTATC	57.4			46127055del
*CDH2*	P1-C1	F: GGGCATGGAGGGAATTTAGGT	57.3	105/119	Intron 1	
		R: TTCAAATGTTTCTGTTCACCATGAC	53.3			
	P2-C2	F: GAGAACAGATGAGATGGCTCTGA	56.1	115/105	Intron 15	
		R: ATCCTTGGGAGAGAGTGTGACTA	56.7			
	P3-C3	F: TTTGGATCTGGTGACTTGTTTG	52.8	93/85	3${}^{\prime }$ UTR	
		R: TTCTCAATGCAACTCCAGGAC	54.9			
	P4-C4	F: TGCATTGATAAAGTTGGAGACTGGT	56.0	105/99	5${}^{\prime }$ UTR	
		R: GCTAATCCCAGATTCCCAATCCA	56.8			
*KIF1A*	P1-K1	F: ACCGAAGACCCCGCTTG	58.6	157/177	3${}^{\prime }$ UTR	
		R: GTCCTGGTGCCCTTGAAC	56.7			
	P2-K2	F: GAACCTTCTGGGCTGACCG	59.2	283/254	Intron 1	
		R: TCTGTGGGAGGACACGCAG	60.4			
	P3-K3	F: ACTCTCTTCCAAACTCTTGCC	54.6	162/180	Intron 1	
		R: CCAAGTCCTCGAACAGGTGA	57.0			
	P4-K4	F: TTGGATGAGCTATGTCGCCT	56.0	103/116	Intron 1	
		R: GCAGGGCTGGGGTCAATC	60.0			

Polymerase chain reaction (PCR) amplification was performed as previously reported (Jin et al., 2016; Li et al., 2017; Xu et al., 2018; Ju et al., 2020), and products were separated on 3.5 % agarose gels. PCR products were sequenced when each primer pair and had different genotypes (Yang et al., 2016).

### 2.4 Amplification and genotyping

According to the mutation frequency and sample size of InDel loci, we used the pooling method to determine 11 pairs of primers and identified one locus (*DYRK2*-P3-11 bp) in XJBC (Table 1). PCR was carried out in a total reaction mixture of 13 µL, consisting of 6.5 µL 2× MIX (i.e. 2× PCR Taq MasterMix) (TsingKe, Xi'an, China), 0.3 µL each primer (forward and reverse primers), 0.3 µL genomic DNA, and 5.6 µL ${\mathrm{ddH}}_{2}O$ (double-distilled water).

We used the Touchdown PCR program as follows: initial denaturation for 4 min at 95 ${}^{\circ }$C, denaturation for 30 s at 94 ${}^{\circ }$C, cycling 18 times; an annealing step for 30 s at 68 ${}^{\circ }$C (1 ${}^{\circ }$C reduction per cycle) with extension for 1000 bp min${}^{-1}$ at 72 ${}^{\circ }$C; another 30 cycles (Czarnik et al., 2009; K. Wang et al., 2020); cooling to 4 ${}^{\circ }$C; and detection of PCR products by 1.5 % agarose gel electrophoresis.

### 2.5 Statistical analyses

We used the chi-square test ($\chi^{2}$) to determine whether SNP variation was in Hardy–Weinberg equilibrium (HWE) (Pan et al., 2013). Furthermore, we used SPSS software (version 18.0) to analyze variance (ANOVA) and the independent sample T test to explore the correlation between InDel loci and milk traits (e.g. 305M (kg)). When necessary, we performed Bonferroni corrections for multiple comparisons (X. Y. Wang et al., 2018). Non-parametric (Kruskal–Wallis) tests in SPSS (version 18.0) were used to analyze data when homogeneity of variances did not follow a normal distribution. P<0.05 was considered statistically significant. In addition, correlations between these traits and polymorphic loci were analyzed (Peng et al., 2019).

Finally, association analyses between milk traits and InDel loci were carried out. The general linear model was as follows:

1 $$Y_{ijk}=\mu +G_{i}+P_{j}+e_{ijk},$$

where $Y_{ij}$ is the phenotypic value of milk traits, μ is the overall population mean, $P_{j}$ is the fixed effect of parity, $G_{i}$ is the fixed effect of genotype, and $e_{ijk}$ is the random error.

## 3 Results

### 3.1 Identification of *DYRK2, CDH2*, and *KIF1A* gene InDel polymorphisms

**Figure 1:**
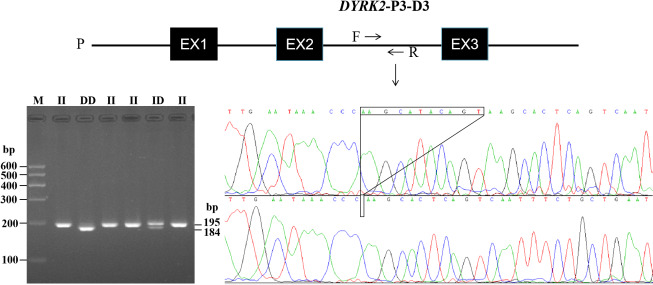
The electrophoresis diagrams and sequence diagrams of InDel locus of bovine *DYRK2* gene. Note that P represents promoter region and EX represents exon.

One InDel locus in *DYRK2* was identified in XJBC, and it had different genotypes (II, ID, and DD) (Fig. 1). Specifically, there was an 11 bp deletion at intron 2 (*DYRK2*-P3-11 bp) (Table 1). Every InDel polymorphism had two or three distinct genotypes: a longer DNA fragment indicating genotype insertion–insertion (II), a shorter DNA fragment indicating genotype deletion–deletion (DD), and two or three (homoduplex) bands indicating genotype insertion–deletion (ID). The sequence diagrams for this InDel are shown in Fig. 1.

### 3.2 Genetic parameters calculation

We listed the frequency of population parameters for this InDel and found that the I allele (0.690) of *DYRK2*-P3-11 bp was more frequent than the D (0.310) allele (Table 2). Parameter analyses showed that the PIC for this InDel was 0.336, indicating moderate genetic diversity (0.25<PIC<0.5).

**Table 2:** Frequencies of genotypes and alleles and diversity parameter for InDel of DYRK2 gene in cattle.

Populations	Sizes	Genotypic			Allelic		HWE	Population			
		frequencies			frequencies		P values	parameters			
		II	ID	DD	I	D		Ho	He	Ne	PIC
XJBC	388	0.493	0.393	0.113	0.690	0.310	0.115	0.572	0.428	1.748	0.336
WLC	50	1.000	0	0	1.000	0	0	1.000	0	1.000	0

Note that HWE represents Hardy–Weinberg equilibrium, Ho represents homozygosity, He represents heterozygosity, Ne represents effective allele number, and PIC represents polymorphism information content.

### 3.3 Novel InDel polymorphisms had significant relationships with milk traits

Next, we studied the correlation between this XJBC *DYRK2* InDel and milk traits. The InDel locus showed a significant relationship with milk traits (Table 3), such as 305M (P<0.05), PY (P<0.05) in the fourth parity, and FY (P<0.05) in the sixth parity.

**Table 3:** Relationship between InDel polymorphism of *DYRK2*-P3-D3 locus and milk traits of XJBC.

Parity	Sizes	Milk traits	Observed genotypes (LSM${}^{a}$ ± SE)			P values
			II	ID	DD	
4	108	305M	${}^{b}4186.32\pm 886.46$	${}^{b}3758.74\pm 1164.28$	${}^{a}4529.67\pm 1313.42$	0.040
	84	PY	${}^{b}146.42\pm 30.63$	${}^{b}136.35\pm 40.00$	${}^{a}173.05\pm 47.32$	0.021
6	20	FY	${}^{a}211.30\pm 70.13$	${}^{b}145.85\pm 45.24$	${}^{b}153.37\pm 43.78$	0.028

Note that 305M represents 305 d milk yield, FY represents fat yield, SCS represents somatic cell score, FP represents fat percent, PY represents protein yield, and PP represents protein percent. The values with different letters (a and b) within the same row are significant at P<0.05 and P<0.00, respectively.

## 4 Discussion

In recent years, InDel makers have been widely used in MAS-based animal breeding (Wang et al., 2019; Z. Wang et al., 2020). InDel mutations of some candidate genes can be used to select excellent phenotypic traits of cattle (Cui et al., 2018; Han et al., 2018). In a previous study, we found that InDel variation in some candidate genes can be used to choose better phenotypic traits in cattle (Wang et al., 2017; Kang et al., 2019). We also found that *DYRK2* was closely associated with milk traits in XJBC (Zhou et al., 2019).

*DYRK2* is a part of the CMGC group of protein kinases. It contains a conservative kinase domain structure and a neighboring N-terminal *DYRK* homology (DH) box. Regulation irregularities in this gene may be related to the occurrence of human breast cancer (Mimoto et al., 2013; Enomoto et al., 2014). This gene has been confirmed to be involved in the development of bovine mammary glands (Tolleson et al., 2017). *DYRK2* is located on chromosome 5 (BTA5), and the 11 bp InDel that we identified was significantly correlated with milk traits in XJBC.

**Figure 2:**
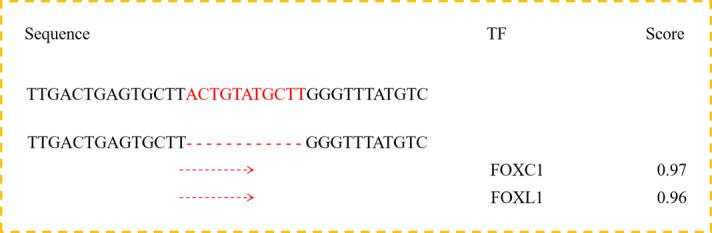
Bioinformatics predict transcription factor binding sites on the bovine *DYRK2* gene 11 bp InDel sequences.

There have been some studies on *DYRK2*, but InDels are often neglected, particularly in introns and UTR (untranslated region) mutations (Maddika et al., 2009; Taira et al., 2012; Yang et al., 2016; Ju et al., 2020). Here, we identified InDel in introns, and there were insertion and deletion mutations as well (Hu et al., 2011; Liu et al., 2015). To the best of our knowledge, this is the first identification of a candidate gene, *DYRK2*, associated with milk traits in XJBC. The PIC value of *DYRK2* was less than 0.4, indicating low genetic diversity. In addition, the InDel variant genotypes and allele distributions in XJBC and WLC were significantly different (P<0.05 or P<0.01) due to their genetic background. XJBC live in Xinjiang Province (northwestern China) (Lin et al., 2010; Li et al., 2018), and WLC live in Shandong Province (eastern China) (Wang et al., 2015). In addition, the genetic diversity of these cattle may differ because they have been subjected to different long-term artificial selection processes.

We found a significant correlation between the identified InDel and milk traits in XJBC, including 305M (P=0.040), PY (P=0.021) in the fourth parity, and FY (P=0.028) in the sixth parity. Correlation analyses confirmed these results. The minor-allele frequency (MAF) value of *DYRK2-*P3-11 bp was low (0.310). In addition, the InDel showed moderate polymorphism (PIC value ≥ 0.3) in both cattle; therefore, *DYRK2*-P3-11 bp (PIC value = 0.336) should be further studied.

According to previous research, intron mutations may affect the host genes factors and interaction between transcription (Van et al., 2003; Fushan et al., 2009). Therefore, we used the online software Jaspar Genereg (http://jaspar.genereg.net/matrix/MA0106.3/, last access: 10 July 2019) to predict transcription factor binding sites in the 11 bp InDel sequence. Bioinformatics analyses showed that forkhead box C1 (*FOXC1*) and forkhead box L1 (*FOXL1*), as transcription factors, could bind to the sequence in the absence of the 11 bp nucleotides (Fig. 2). This highlights a possibility that *FOXC1* and *FOXL1* influence milk traits in cattle. At the same time, *FOXC1* and *FOXL1* encode members of the forkhead and/or winged-helix box (FOX) family of transcription factors, and FOX transcription factor has a unique DNA-binding forkhead domain that plays a key role in regulating multiple processes including gene expression (Masuko et al., 2004) and cell proliferation. However, the effects of our identified InDel on *FOXC1* and *FOXL1* factor-induced milk traits have not been determined, and further studies are needed.

## 5 Conclusion

Briefly, an InDel in the *DYRK2* candidate gene was significantly correlated with milk traits in XJBC. Our findings enrich the data on the genetic diversity of *DYRK2* genes in cattle. Potentially useful DNA markers can be identified and used for MAS-based cattle breeding.

## Data Availability

The data from this study can be accessed from the authors upon a reasonable request.

## Appendix A Abbreviations

**Table:**

*CDH2*	cadherin 2
*DYRK2*	dual-specificity tyrosine phosphorylation regulated kinase 2
*KIF1A*	kinesin family member 1A
GWASs	genome-wide association studies
InDel	insertion/deletion
MAS	mark-assisted selection
XJBC	Xinjiang Brown cattle
WLC	Wagyu × Luxi crossbreed cattle
305M	305 d milk yield
CNV	copy number variation
FP	milk fat percentage
FY	milk fat yield
PP	milk protein percentage
PY	milk protein yield
SCS	somatic cell score
SNP	single-nucleotide polymorphism
HWE	Hardy–Weinberg equilibrium
Ho	homozygosity
He	heterozygosity
Ne	effective allele numbers
PIC	polymorphism information content
UTR	untranslated region
LSM	least square estimate
